# Mental Health and Well-Being Among Home Health Aides

**DOI:** 10.1001/jamanetworkopen.2024.15234

**Published:** 2024-06-06

**Authors:** Melissa Yanez Hernandez, Elizabeth Fong-Chy Kuo, Yefrenia Henriquez Taveras, Ann Lee, Aida Ramos, Joanna Ringel, Susan Andreae, Emma Tsui, Monika M. Safford, Ariel C. Avgar, Megan J. Shen, Nicola Dell, Daniel Shalev, Catherine Riffin, Faith Wiggins, Elissa Kozlov, Nathalie Moise, Madeline R. Sterling

**Affiliations:** 1 Division of General Internal Medicine, Department of Medicine, Weill Cornell Medicine, New York, New York; 2 1199SEIU Training and Employment Fund, New York, New York; 3 Kinesiology Department, University of Wisconsin-Madison; 4 School of Public Health, City University of New York, New York; 5 School of Industrial and Labor Relations, Ithaca, New York; 6 Clinical Research Division, Fred Hutchinson Cancer Center, Seattle, Washington; 7 Cornell Tech, New York, New York; 8 School of Public Health, Rutgers University, New Brunswick, New Jersey; 9 Columbia University Irving Medical Center, New York, New York

## Abstract

**Importance:**

Home health aides and attendants (HHAs) provide essential care to older adults and those with chronic conditions in the home. However, some HHAs struggle with poor mood and stress, which may have been exacerbated by the COVID-19 pandemic.

**Objective:**

To elicit HHAs’ perspectives toward mental health and well-being, including how their job influences both and how to better support the workforce in the future.

**Design, Setting, and Participants:**

For this qualitative study, focus groups and interviews with HHAs were facilitated in English and Spanish from August 17, 2022, to February 9, 2023, in partnership with the 1199SEIU Training and Employment Fund, a benefit fund of the 1199SEIU United Healthcare Workers East and the largest health care union in the US. Included were HHAs at risk for poor mental health and well-being, which were defined as having at least mild or more symptoms on either the 8-item Personal Health Questionnaire depression scale, the 4-item Cohen Perceived Stress Scale, or the University of California, Los Angeles Loneliness Scale.

**Exposure:**

Mental health and well-being of HHAs.

**Main Outcomes and Measures:**

Focus groups and interviews were audio recorded, professionally transcribed, and translated. A thematic analysis was performed that was informed by Pender’s Health Promotion Model and the National Institute for Occupational Safety and Health’s Total Worker Health model.

**Results:**

A total of 28 HHAs from 14 different agencies participated (mean [SD] age, 54.3 [10.8] years; 26 female [93%]). Seventeen participants (61%) spoke Spanish at home. Five key themes emerged: (1) HHAs’ attitudes toward mental health and well-being were influenced by a variety of personal and cultural factors; (2) HHAs’ relationships with their patients impacted their mood in both positive and negative ways; (3) structural and organizational aspects of the job, alongside the COVID-19 pandemic, impacted HHAs’ mood and stress levels; (4) HHAs used a variety of strategies to cope with their emotions; and (5) HHAs were eager for interventions that can improve their mood, particularly those that bring them closer to their colleagues.

**Conclusions and Relevance:**

These findings suggest that HHAs’ mental health and well-being may be influenced by both personal and occupational factors. Interventions and policies to better support their emotional well-being on the job are warranted.

## Key Points

**Question:**

What are home health aides’ and attendants’ (HHAs’) perspectives toward mental health and well-being, and how does their job influence them?

**Findings:**

In this qualitative study of 28 HHAs employed by 14 different home care agencies, participants reported various personal and occupational factors that affected their mental health and well-being, particularly their relationships with patients. While the participants used multiple strategies to cope, they were eager for support to help manage mood and stress on the job.

**Meaning:**

These findings suggest that interventions and policies geared toward mental health are needed to better support HHAs as they provide essential patient care.

## Teaser

This qualitative study investigated home health aides’ perspectives toward mental health and well-being and how their job influences these areas of life.

## Introduction

Most older adults prefer to stay in their homes as they age and to avoid nursing homes, a concept known as aging in place.^1^ To age in place, many individuals require help at home. While this help is often provided by family caregivers, home health aides and attendants (HHAs) are increasingly providing assistance.^2,3^ These HHAs are a rapidly growing workforce trained and certified to provide personal and medical care, as well as emotional support, in the home.^3^ Unlike other health professionals, HHAs are with patients in the home for long periods, which gives them a unique vantage point to observe, support, and advise patients.

Despite their contributions to patient care, recent studies have found that HHAs themselves are a vulnerable workforce susceptible to poor mental health.^4,5^ Much of this susceptibility may partly stem from HHAs’ marginalized positioning in the health care industry and historically poor labor protections as a workforce.^6,7^ Additionally, HHAs, who are mostly women and people from minoritized racial and ethnic groups, are paid dismally low wages, and 26% lack health insurance, which inhibits their access to quality mental health care.^8^ The COVID-19 pandemic worsened HHAs’ working conditions and their physical and mental health.^9,10^ Home health aides and attendants, who often work alone without coworkers, provided care during the COVID-19 pandemic without the personal protective equipment and other basic supports that institutionally based workers received (eg, paid sick leave and access to testing).^9,10^ This resulted in many living with fear of contracting and transmitting COVID-19.^9^ A survey of agency-employed HHAs in New York City found that two-thirds were struggling to manage their mental health post pandemic.^11^ Overall, since COVID-19, 70% of HHAs wanted more support to cope with stress, anxiety, and mood.^12^ Left untreated, HHAs’ poor mental health may threaten not only their well-being but also their ability to provide high-quality patient care.

While prior studies have quantified the prevalence of HHAs’ poor mental health,^4^ the unique workplace stressors in the home environment during COVID-19, and their desire for support, there has been little investigation with HHAs themselves about what factors influence their perceptions of mental health and how their job contributes, particularly in the post–COVID-19 era. Additionally, while a recent study elicited home care agency leaders’ perspectives for how to address the workforce’s deficits in mental health and well-being,^13^ HHAs have rarely been asked to provide their own recommendations on potential solutions, a critical aspect of sustainable and effective intervention development.

Thus, we aimed to understand HHAs’ attitudes toward mental health and well-being and how their job creates barriers and/or facilitators toward maintaining them. Additionally, we elicited HHAs’ perspectives on what types of interventions could meet their emotional needs in the context of their job.

## Methods

### Setting and Study Population

This qualitative study was conducted virtually from August 17, 2022, to February 9, 2023, in New York City in partnership with the 1199SEIU Training and Employment Fund (TEF), a labor management fund of the 1199SEIU United Healthcare Workers East, the largest health care union in the US.^14^ 1199SEIU TEF provides education and training to more than 55 000 HHAs employed by more than 50 licensed and certified home care agencies in New York.^15^ While not all HHAs in New York are employed by unionized agencies, nearly half are, which made 1199SEIU TEF an important access point for this study.^16^ The study protocol was approved by the BRANY (Biomedical Research Alliance of New York) institutional review board (No. 22-08-234-380). All participants provided electronic informed consent, and each received a $50 gift card for their time. The study followed the Consolidated Criteria for Reporting Qualitative Research (COREQ) reporting guideline.

To ensure that we focused on HHAs most in need, we included those at risk for poor mental health and well-being as defined in the literature and with input from experts in long-term care. To be eligible for this study, HHAs (1) spoke English or Spanish; (2) were employed by a licensed or certified home care agency in New York City; and (3) had 1 or more risk factors for poor mental health and well-being assessed across 3 domains, including depressive symptoms, stress, and loneliness. Depressive symptoms were assessed with the 8-item Personal Health Questionnaire depression scale^17^; a score of 5 or higher on a scale from 0 to 24 was considered positive for mild depressive symptoms.^18,19^ Stress was assessed using the 4-item Cohen Perceived Stress Scale^20^; a score of 6 or higher on a scale of 0 to 16 was considered positive for moderate or greater stress symptoms.^21^ Loneliness was assessed using the 3-item University of California, Los Angeles Loneliness Scale^22^; a score of 6 or higher on a scale of 3 to 9 was considered positive for loneliness.^23^ These validated assessments were administered in English and Spanish.

Using a standardized recruitment script, 1199SEIU TEF staff reached out to affiliated HHAs who were in contact with the organization during the study period. If interested in participating, Weill Cornell Medicine research assistants (M.Y.H. and E.F.-C.K.) then sent an electronic screening survey via REDCap to HHAs to assess eligibility.^24,25^ If eligible, HHAs were scheduled for a language-concordant virtual focus group conducted through videoconferencing. Due to scheduling constraints among HHAs (ie, patient shifts, commutes), interviews were used as a substitute form of data collection when needed.

### Data Collection

Two researchers (M.Y.H. and E.F.-C.K.) trained in qualitative research methods moderated the 60- to 90-minute virtual focus groups and conducted the interviews using a semistructured topic guide. The topic guide was informed by a previously published conceptual framework that combined aspects of Pender’s Health Promotion Model and the National Institute for Occupational Safety and Health’s Total Worker Health conceptual model (eFigure in Supplement 1).^5^

The topic guide asked participants about their overall health and specifically about their attitudes toward mental health and well-being, including how their job influences their mood and stress levels. Participants were also asked about the challenges and facilitators that their work presents to their overall mental health and well-being, including the COVID-19 pandemic. Additionally, they were asked about their preferences toward future interventions that could meet their needs (eAppendix in Supplement 1).^5,26,27^ In addition to the interviews and focus groups, self-reported demographic characteristics, including age, sex, race (including Asian, Black, White, and other [including Hispanic, Brown, or not specified]) and Hispanic or Latinx ethnicity, educational level, and employment history, were collected. Race and ethnicity are essential to include since HHAs are historically a minoritized population (mostly women and women of races and ethnicities other than White) of frontline health care workers; we wanted to ensure that we had diversity in race and ethnicity and by language spoken.

### Data Analysis

Interviews and focus groups were conducted via videoconferencing, audio recorded, and transcribed professionally. Data were organized using a custom-built Python-based visualization tool, which has been previously used in other qualitative investigations.^9,28^ We followed a thematic analysis approach to iteratively develop a codebook that included framework-driven, a priori codes and emergent, inductive codes.^29,30,31,32^ First, 2 investigators (M.Y.H. and E.F.-C.K.) trained in qualitative coding independently reviewed 5 transcripts and created codes. The investigators met to reconcile and consolidate their codes to create an initial codebook with oversight by a third investigator (Y.H.T.) and a senior investigator (M.R.S.). The codebook was then applied to the remaining transcripts, pausing at every fifth transcript to review, reconcile, and consolidate as a team. The codes were categorized into themes and subthemes by the investigative team. Focus groups and interviews concluded when data saturation, the point at which no new themes emerged, was reached.^33^ We examined data across focus groups and interviews to assess for differences in results from these 2 interview modalities but found the data to be thematically consistent. Thus, data were merged and presented as 1 dataset regardless of data collection modality.^34,35^ Descriptive statistics were performed to characterize the sample. We performed the data analyses using Stata/MP, version 14 software (StataCorp LLC).

## Results

### Participant Characteristics

A total of 28 HHAs employed by 14 distinct home care agencies in New York City participated; 19 participated across 4 focus groups, while 9 were interviewed individually. Together, their mean (SD) age was 54.3 (10.8) years; 26 were female (93%) and 2 (7%) male; 1 identified as Asian (4%), 11 as Black (39%), 3 as White (11%), and 13 as other race (46%); and 16 identified as Hispanic or Latinx (57%) (Table 1). They had worked as HHAs for a median of 16.5 years (IQR 7.0-24.0 years). Seventeen participants (61%) spoke Spanish at home. Overall, 17 participants (61%) had at least mild depressive symptoms and 7 (25%) had at least moderate depressive symptoms, 27 (96%) reported feeling stressed, and 12 (43%) reported feelings of loneliness. Of the 28 participants, 10 (36%) had 1 or more risk factors for poor mental health and well-being, 8 (28%) had 2 risk factors, and 10 (36%) had all 3 risk factors (Figure).

**Table 1.** Sample Characteristics

Characteristic	No. (%)
No. of respondents	28
Age, mean (SD), y	54.3 (10.8)
Sex	
Female	26 (93)
Male	2 (7)
Race	
Asian	1 (4)
Black	11 (39)
White	3 (11)
Other^a^	13 (46)
Ethnicity	
Hispanic or Latinx	16 (57)
Highest level of education	
No degree or some high school	5 (18)
Completed high school or GED	9 (32)
Some college	8 (29)
College degree	5 (18)
Graduate degree	1 (4)
Foreign born	25 (89)
Primary language spoken at home	
English	8 (29)
Spanish	17 (61)
Other	3 (11)
Duration of employment as an HHA, median (IQR), y	16.5 (7.0-24.0)
Job satisfaction, mean (SD)^b^	18.2 (6.0)
Discrimination in the workplace^c^	4 (14)
Perceived social support score, mean (SD)^d^	56.1 (16.2)
MCS score, mean (SD)^e^	48.6 (9.14)
PCS score, mean (SD)^f^	49.3 (7.2)

Abbreviations: GED, General Educational Development; HHA, home health aide and attendant; MCS, Mental Component Summary of the 12-Item Short Form Survey; PCS, Physical Component Summary of the 12-Item Short Form Survey.

^a^ Participants who selected other race identified as Hispanic or Brown or did not specify.

^b^ Overall experience in the workplace was measured by the Work Domain Satisfaction Scale. Scores range from 5 to 30, with higher scores indicating higher job satisfaction.

^c^ Discrimination in the workplace was measured by the National Institute of Occupational Safety and Health Well-Being Questionnaire. An answer of yes to any of the 4 questions indicated discrimination in the workplace.

^d^ Social support was measured by the Multidimensional Scale of Perceived Social Support. Total scores range from 7 to 84, with higher scores indicating a greater degree of perceived social support.

^e^ A score of 42 or less may be indicative of clinical depression.

^f^ A score of 50 or less is the recommended cutoff for determining a physical condition.

**Figure.**
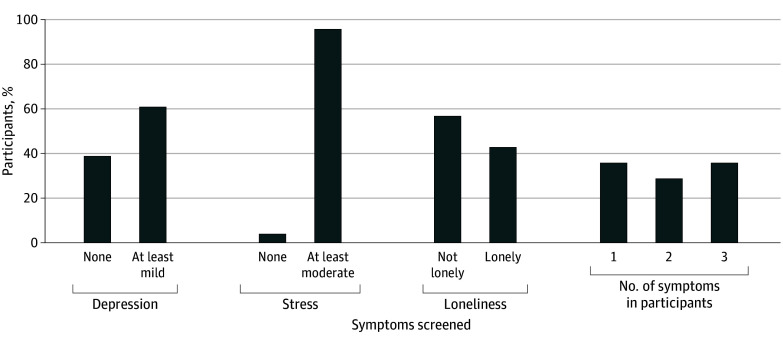
Percentage of Study Sample (N = 28) With Self-Reported Depressive Symptoms, Perceived Stress, or Loneliness Depressive symptoms were assessed using the 8-item Personal Health Questionnaire depression scale, for which scores of 5 or higher on a scale of 0-24 are considered positive for depressive symptoms.^17^ Stress was assessed using the 4-item Cohen Perceived Stress Scale, with scores of 6 or higher on a scale of 0-16 indicating more perceived stress.^21^ Isolation was assessed using the 3-item University of California, Los Angeles Loneliness Scale, with scores of 6 or higher on a scale of 3-9 indicating loneliness.^22^

### Major Themes

Overall, 5 major themes emerged. Themes and subthemes are outlined in Table 2 and are detailed below, along with illustrative quotes.

**Table 2.** Themes and Subthemes

Theme	Description	Subthemes
1	HHAs’ attitudes toward mental health and well-being may be influenced by a variety of personal and cultural factors.	Cultural factors, including stigma Role as a family caregiver Personal experiences with health
2	HHAs’ relationships with their patients may influence their mood in both positive and negative ways.	Interactions with patients Patient’s family dynamics
3	The structure and organizational aspects of the job, alongside the COVID-19 pandemic, may contributed to HHAs’ mood and stress levels.	Compensation and benefits Communication challenges and lack of integration and support Stress associated with COVID-19, including risk of exposure and safety Continuing to work, despite occupational challenges and health risks
4	HHAs may use a variety of strategies to cope with their emotions.	Health behaviors and techniques to cope in general Strategies used to cope on the job
5	HHAs may be eager for interventions and programs that can improve their mood.	Desire for information and knowledge Interest in peer support programs

Abbreviation: HHA, home health aide and attendant.

#### Theme 1: Influence of Personal and Cultural Factors on HHAs’ Attitudes Toward Mental Health and Well-Being

The participants’ attitudes toward mental health and well-being were notably influenced by cultural and personal factors. While some participants reported feeling comfortable with discussing their mood, others reported stigma around mental health, which made it challenging. One participant said that “a person mentions something about mental health, then it crosses your mind that this person may...have mental problems. The first thing you think is, ‘they’re crazy’.…It makes you feel ashamed or afraid.”

In general, personal responsibilities outside of work, including family caregiving, affected their mood. As 1 participant explained, “I have personal stress that I’m going through with my young son, and sometimes it’s emotional. I come to work, and I have to put that to the side and to do my job in a professional way. Sometimes it’s hard.”

While participants talked about caring for others, they also reported needing to manage their own medical conditions, including diabetes and hypertension, which added to their stress. One participant explained, “I have diabetes, and I worry a lot about it—what I eat, how much I exercise, my weight.…”

#### Theme 2: Influence of Relationships With Patients on HHAs’ Mood

Participants reported that their relationships with patients affected their mood in both positive and negative ways. Certain situations were particularly challenging, including caring for patients who require additional services beyond the agency’s plan of care (ie, housekeeping or extended hours) or caring for those with complex or emotionally taxing illnesses (dementia, depression, etc). One participant recounted, “Well, with the job, of course you’re working with [patients]...they have their little mood and their little attitude and tend to get you....I try to be always with a smile, even though it hurts me.”

Another participant described that at times, patients could be racist toward them, which presented a problem since they are in the home alone with the patient for long periods. One participant said, “Some [patients] are racists, and they look at you as if you are less than them, but I don’t pay that any mind. I do my job and if it ends up being hard, well, I tell them to find me another.”

Patients’ family members could also be problematic. As 1 participant explained, “They live with family, and there are some that are hateful. What you do is to try to focus on your work and not pay them much attention…and it is bad to arrive at a house where you feel uncomfortable.”

Counter to the negatives, other participants reported a deep sense of gratification and joy from their work. They discussed how forging meaningful relationships with their patients made them feel good. Many participants reported taking pride in their work, often driven by their desire to make a positive difference in the lives of their patients. When speaking about her work, 1 participant stated, “I love working in the health care field. I love taking care of people. I love people to be happy. I love to give all what I have to them, try to do my best.”

#### Theme 3: Influence of Structural and Organizational Aspects of the Job and COVID-19 on HHAs’ Mood and Stress Levels

Several participants described how the structural and organizational aspects of their job, alongside the COVID-19 pandemic, contributed to their stress levels. Many described that working extended hours, having long commutes, and receiving inadequate compensation substantially impacted their well-being. As 1 participant explained, “[The] cost [of living] is very high right now....Nobody can live with $17 no more in New York. This is another stress.” Many participants also reported having insufficient time for themselves, including taking breaks or even having lunch, which compounded the strain.

Additionally, participants highlighted several issues pertaining to their home care agencies, including poor communication and insufficient information regarding their patients, that contributed to stress on the job. For example, some participants mentioned not being told about their patients’ health issues both prior to and during home care episodes, often having to rely on family members to obtain relevant details. As 1 participant explained, “Some of the clients…don’t even know what is their health problem. They just hire us and send us out on these cases. They don’t know…somebody’s family members don’t even discuss it.”

Furthermore, participants reported that lack of explicit guidelines and information from the agency to the client regarding how to respect HHA boundaries created challenges. A participant recounted, “it’s a lot of challenges being a home attendant. If God bless you, you have good clients that treat you like your own, you’re blessed. A lot of people treat home attendants as a domestic staff. They treat them like a slave.”

Many participants described the challenges of working during and since the COVID-19 pandemic and how it affected their well-being. One participant stated, “I thought I was losing my mind….I didn’t stay home. I just washed my hands, cleaned like they told us to do…at that time they gave me a 12-hour, 6-day case, and I never worked so much in my life, but I had prayed and I had prayed for help, and that was my help.”

Despite considerable stress and concern, participants acknowledged persisting in their work with their patients. One HHA stated, “we never stopped working, I mean, I always said we have to do what we have to do as citizens, because nobody wanted to work, everybody was hiding, and…we were normal.”

#### Theme 4: Variety of Strategies to Cope With Emotions

Participants reported using a range of strategies and health behaviors to manage their emotions and mood both off and on the job. When asked how they cope, many discussed doing breathing exercises and meditation, engaging in prayer, and physical activity. As 1 participant explained, “The first thing I do is pray to God to give me strength to move forward because the struggle here has not been easy.”

Additional strategies that participants reported using included listening to music, watching TV, playing games, reading, and taking courses. One participant said, “Sometimes I try to hide it, but I do cry. But then afterwards, I try to calm down a bit, I don’t know, sometimes I play music. I entertain my mind.”

Beyond these activities, many participants compartmentalized difficult work situations to help get through the day. As 1 participant explained, “I try not to make my emotions get to my job….I’m there to take care of her. I do what I got to do then…deal with whatever I got to deal with later.”

Some participants did not just compartmentalize patient dynamics but also tried to leave stress from their personal lives, including family caregiving responsibilities, at home during their shift. Notably, although many participants reported that the job contributed to stress, some perceived their work as a means of escaping from problems at home. They expressed a sense of anticipation for their work schedule since this could help them cope with their experiences at home. As 1 participant remarked, “When I’m working, I’m in a good mood. This is my escape.”

#### Theme 5: Eagerness for Interventions That Can Improve Their Mood

Many participants wanted programs and supports that could improve their mood and feelings on and off the job. One participant explained, “I wish I could participate in a program that could make me feel good, I mean, that could help me.”

Participants reported that this type of information would benefit their health and their patients. Many suggested that courses on mental health, or wellness in general, from their union or their home care agencies would be beneficial. One participant said, “I really like to learn…you can share that [knowledge] with other people too.”

Participants also wanted to learn from each other. They reported that peer coaching, which they have experienced before, could be a potential solution, particularly for mental health where stigma and occupational stressors may be influential. They appreciated how it offers a way to talk with fellow HHAs who had similar job-related challenges and learn from and support each other in a nonjudgmental way. One participant stated, “It is always good to share different opinions with someone who knows your situation. And to work together too.”

The need for on-the-job support from other HHAs was also driven, in part, by participants’ expressed difficulty in discussing their professional experiences with their families. The reasons they cited were concerns about patient privacy or their preference to keep work and family life separate.

## Discussion

This qualitative study offers findings about HHAs’ attitudes toward mental health and well-being, an issue currently affecting the health and sustainability of the long-term-care workforce.^4,5^ Our findings indicate that while HHAs’ attitudes may be shaped by personal circumstances, their occupation and the unique experience of providing care to people in the home may heavily influence their mood. Notably, the HHA-patient relationship and their workplace environment may influence them in both positive and negative ways. We also found that while HHAs may use a variety of strategies to cope with stress and their emotions, they may desire additional support.

In line with Pender’s Health Promotion Model and the National Institute for Occupational Safety and Health’s Total Worker Health models, it is clear that structural and social determinants (eg, poor agency communication, patients’ and families’ racial biases) may influence HHAs’ personal and professional challenges.^6,7,8^ Unlike other health care professionals, HHAs provide care to patients in the home, which is an inherently less structured, standardized, and regulated workplace compared with clinics, hospitals, or postacute care facilities.^8^ Prior work has found that working in the home may contribute to the perception that HHAs provide less skilled or medically relevant care than other health care professionals.^36^ Our work adds new dimensions surrounding HHAs’ experiences. That is, beyond the known structural challenges, interpersonal dynamics and organizational factors related to the job may influence HHAs’ mental health and well-being. For example, their relationship with a single patient in the home (good or bad) or with that patient’s family could have a lasting impact. Notably, a recent study found that a positive relationship and more connectedness between HHA and patient were associated with higher-rated job satisfaction among HHAs.^37^ Our study offers insight into why this might be the case. A recent study found that HHAs often interact with a variety of family caregivers in the home.^38^ Previously, these interactions were noted to impact caregiving functions and tasks; however, we also found that it may influence HHAs’ mood and stress levels. Additionally, we found that aspects of the job itself (lack of safety, challenging communication practices, etc) influenced participants’ mood and stress which have been reported as worsening during the COVID-19 pandemic.^9,11,27^ This finding is consistent with other recent studies but offers a post–COVID-19 perspective on how these conditions have had lasting outcomes.^39,40,41^

It should be noted that HHAs may be coping with stress and poor mood in a variety of ways. Similar to a recent study by Lam and Baxter,^42^ we found that HHAs create and maintain boundaries between their professional and personal relationships. A key strategy was compartmentalizing, since participants found that it prevented their emotions from compromising patient care. Additionally, some participants, particularly those of Hispanic or Latinx ethnicity, discussed how in difficult times, faith and prayer helped. Regardless of their current strategies, the majority of our participants wanted more support, particularly more information, knowledge, and resources on how to manage their mood and stress. New here is that the HHAs offered suggestions of what programs or interventions would work best for them, including support from trained peers. This finding is consistent with a few studies that found peer coach–delivered emotional support programs were feasible and well-received among HHAs before and during COVID-19.^26,27,43^ Future studies are underway to formally tailor these programs to the needs of HHAs and evaluate their impact HHAs’ mental health and well-being, delivery of quality care, and patient outcomes.

### Implications

Our findings suggest that policies are needed to better support the mental health and well-being of HHAs, particularly those who may be at risk. While some of our findings can inform change at the agency level—for example, ensuring that HHAs and patients have positive working relationships—other solutions require external, large-scale investments. Unlike other place-based health care workforces, HHAs receive most of their support from their agencies, many of which were financially challenged during the COVID-19 pandemic, with additional support from unions and trade organizations.^10^ Moreover, HHAs who are hired directly by patients and family members (ie, Consumer-Directed Personal Assistance Programs) or those in the gray market may have less access to systematic resources. Recent legislation directed toward the HHA workforce, including the Better Care Better Jobs Act and an executive order by the Biden-Harris Administration, may be steps in the right direction.^44^ Although the executive order aims to improve job quality for HHAs through additional Medicaid funding, part of this funding could be designated for mental health services and support.

Beyond policy efforts, additional research is needed to test and implement culturally and occupationally tailored interventions to the workforce. Our findings support the use of peer coaching as a mechanism to teach and support HHAs. A randomized clinical trial tested a peer coach–delivered intervention among HHAs in Oregon and found that it improved workplace safety and health behaviors.^27^ Our study findings suggest that such programs could be expanded to encompass topics related to mood and stress. If successful, such programs could also serve as a career ladder or pathway for HHAs to serve as health coaches, which aligns with current policy initiatives at the national level.^45^

### Strengths and Limitations

Strengths of our study include our partnership with a labor management fund of the largest health care union in the US, which enabled us to recruit a demographically diverse sample of HHAs and employment agencies. We also used 2 established conceptual models to inform the interview topic guide and data analysis. Finally, we collected data on a variety of measures about HHAs’ mental health and well-being using validated scales; although our sample size is small, this work provides new self-reported data on the workforce, which has rarely been collected since the COVID-19 pandemic.

This study also has notable limitations. While we included a diverse sample of HHAs, we did not include nonunionized or non–agency-employed HHAs or HHAs who lived outside of an urban area. Additionally, there are several ways to examine mental health and well-being. While we took a literature and occupation-guided approach, we recognize that other inclusion criteria and questions may also be appropriate to gain additional perspectives.

## Conclusions

The findings of this qualitative study suggest that despite their integral role in patient care, HHAs themselves are a vulnerable workforce. Their mental health and well-being may be influenced by personal and occupational factors, including their relationships with patients. The COVID-19 pandemic exacerbated these challenges and continues to have a lasting impact. Many participants use a variety of coping mechanisms; however, interventions, programs, and policies that could better support the mental health of the HHA workforce are warranted. These interventions should be culturally and occupationally adapted to meet the workforce and home environment needs.

## Notes

Supplement 1. **eAppendix.** Topic Guide for Focus Groups and Interviews **eFigure.** Adapted Conceptual Framework

Supplement 2.
Data Sharing Statement
