# Supplementary material for: Mental Health and Well-Being Among Home Health Aides
Source: JAMA Netw Open. 2024 Jun 6;7(6):e2415234. doi: 10.1001/jamanetworkopen.2024.15234 (PMC11157351; doi:10.1001/jamanetworkopen.2024.15234)
Supplement: Supplement 1. — eAppendix. Topic Guide for Focus Groups and Interviews eFigure. Adapted Conceptual Framework [file jamanetwopen-e2415234-s001.pdf]

Supplemental Online Content

Yanez Hernandez M, Kuo EF-C, Taveras YH, et al. Mental health and well-being among home health aides. *JAMA Netw Open*. 2024;7(6):e2415234.
doi:10.1001/jamanetworkopen.2024.15234

eAppendix. Topic Guide for Focus Groups and Interviews

eFigure. Adapted Conceptual Framework

This supplemental material has been provided by the authors to give readers additional information about their work.

© 2024 Yanez Hernandez M et al. *JAMA Network Open.*

eAppendix. General Topic Guide for Focus Groups and Interviews

Opening Question – General health

1. Do you have any health problems that worry you? Or, put another way, do you have any health problems that you have to manage?

Mental health and well-being, general

2. When you hear "mental health" – what does that mean to you?

3. In general, have you ever or do you currently struggle with your mood? Do you ever feel stressed? Lonely?

Your job as a home health aide or attendant (HHA) and how it influences mental health and well-being

4. Please reflect on your day-to-day work as a HHA. How does your job impact your mood? Stress? Feelings?

   Probe: How does caring for patients/clients in the home impact you and your mental health?

5. How does your job impact your general health? your ability to engage in healthy habits?

6. What do you do when or if you don't feel right? How do you cope when you feel stressed, sad, or upset?

   Probe: Where do you get information about mental health?

   Probe: What makes you feel better? Worse?

Impact of COVID-19

7. Our research team previously spoke with and surveyed hundreds of HHAs during COVID-19. We found that many struggled with mood and stress during the pandemic. We want to know more about this, since COVID-19 has subsided. How would you say the pandemic is impacting your mood these days? (now)

Resources and Attitudes Towards Future Interventions

8. 1199SEIU TEF (your union) offers programs and resources to address the mental health and mood of HHAs, like yourselves. (Ex: Teladoc, phone counseling)

   Probe: Have you used any of these? Why, why not?

   Probe: Have you ever sought the advice of a mental health professional? Why /why not?

© 2024 Yanez Hernandez M et al. *JAMA Network Open.*

9. If you had the opportunity to participate in a health program geared towards mental health, would you be interested?

10. If applicable, tell me about some of the past experiences you have had participating in wellness program(s)?

    Probe: What went well? /What didn't go well?

    Probe: What could have been done to make the experience more beneficial?

11. What types of programs or supports would be of most interest to you?

© 2024 Yanez Hernandez M et al. *JAMA Network Open.*

eFigure. Adapted Conceptual Framework

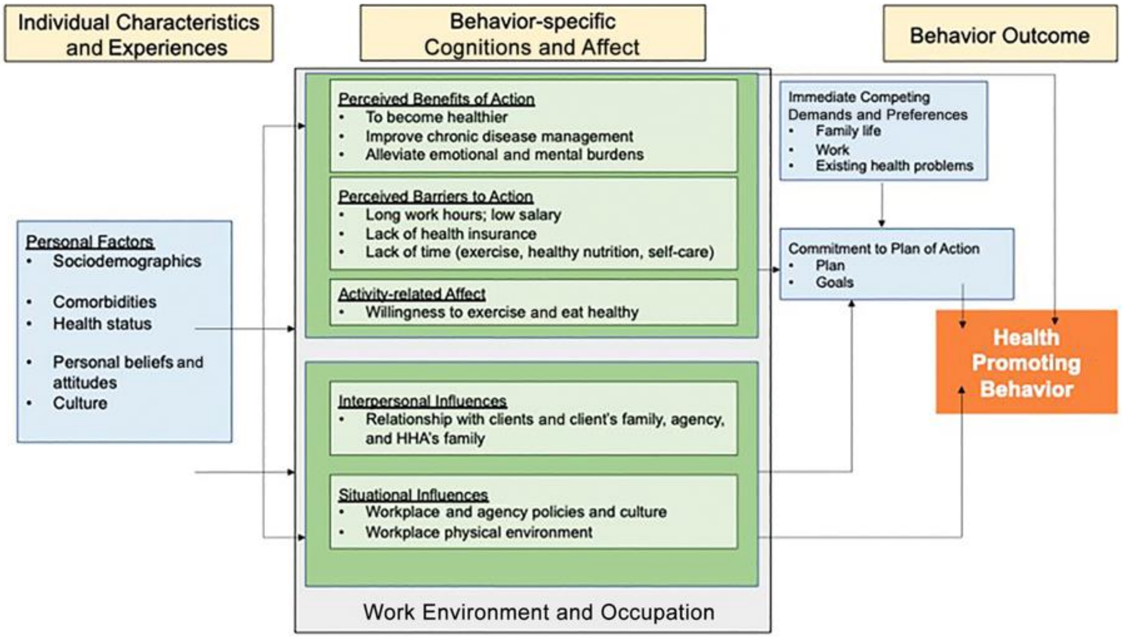

Note. Adapted from the Pender's Health Promotion Model and the National Institute for Occupational Safety and Health's (NIOSH)'s Total Worker Health conceptual Model.

Previously published: Cho J, Toffey B, Silva AF, Shalev A, Safford MM, Phillips E, Lee A, Wiggins F, Kozlov E, Tsui EK, Dell N, Avgar AC, Andreae SJ, Sterling MR. To care for them, we need to take care of ourselves: A qualitative study on the health of home health aides. Health Serv Res. 2023 Jun;58(3):697-704.

© 2024 Yanez Hernandez M et al. *JAMA Network Open.*
