# Supplementary material for: Mental Health and Well-Being Among Home Health Aides
Source: JAMA Netw Open. 2024 Jun 6;7(6):e2415234. doi: 10.1001/jamanetworkopen.2024.15234 (PMC11157351; doi:10.1001/jamanetworkopen.2024.15234)
Supplement: Supplement 2. — Data Sharing Statement [file jamanetwopen-e2415234-s002.pdf]

Data Sharing Statement

Yanez Hernandez. Mental Health and Well-Being Among Home Health Aides. *JAMA Netw Open*. Published June 06, 2024. doi:10.1001/jamanetworkopen.2024.15234

Data
Data available: No
